# Aspects of online college science courses that alleviate and exacerbate undergraduate depression

**DOI:** 10.1371/journal.pone.0269201

**Published:** 2022-06-01

**Authors:** Carly A. Busch, Tasneem F. Mohammed, Erika M. Nadile, Katelyn M. Cooper

**Affiliations:** Research for Inclusive STEM Education Center, School of Life Sciences, Arizona State University, Tempe, Arizona, United States of America; Satyawati College (Eve.), University of Delhi, INDIA

## Abstract

Depression is a top mental health concern among college students, yet there is a lack of research exploring how online college science courses can exacerbate or alleviate their depression. We surveyed 2,175 undergraduates at a large research-intensive institution about the severity of their depression in large-enrollment online science courses. The survey also explored aspects of online science courses that exacerbate or alleviate depression and we used regression analyses to assess whether demographics predicted responses. Over 50% of undergraduates reported experiencing depression and LGBTQ+ students, financially unstable students, and lower division students were more likely to experience severe rather than mild depression compared to their counterparts. Students reported difficulty building relationships and struggling to perform well online as aspects of online science courses that exacerbated their depression and the flexible nature of online courses and caring instructors as aspects of online courses that alleviated their depression. This study provides insight into how instructors can create more inclusive online learning environments for students with depression.

## Introduction

At least a quarter of undergraduates are estimated to experience depression, making it one of the most common mental health concerns among college students [1]. Depression, defined as consistent feelings of unhappiness, hopelessness, and a loss of motivation or interest in actions that an individual previously enjoyed [2], has been correlated with decreased social integration [3], poor academic performance [4–6], and increased attrition among undergraduates [7, 8]. As a result, colleges and universities are increasingly recognizing the need to improve undergraduate mental health, specifically the awareness of mental illness and access to resources [9–12]. While the majority of efforts have focused on increasing student access to campus mental health services [10, 11], an emerging approach is to identify aspects of education that can exacerbate students’ depression in an effort to change educational environments to be more inclusive of students with depression and/or help students with depression cope with such stressors [13–15].

Science courses in particular may present challenges for individuals with depression. Reports of chilly, unwelcoming, and competitive science course environments that are considered ‘neutral’ or devoid of social influence [16–18] are predicted to exacerbate students’ depressive symptoms [14, 19–22]. However, no studies have examined the frequency and severity of depression specifically among science students. Further, depression is known to affect groups that are traditionally underrepresented or underserved in science including women [23], first-generation college students [24], LGBTQ+ individuals [25], students from low socioeconomic backgrounds [25], and students with disabilities [26, 27]. Notably, students report that depression negatively affects their abilities to learn science, namely by hindering cognitive functions such as attention and time management, language skills, executive functions, and problem solving [28, 29]. As such, understanding what specific aspects of college science courses affect depression, whether such aspects disproportionately affect depression among individuals who identify within marginalized groups, and how to potentially alleviate depressive symptoms may be effective steps toward improving student performance and establishing a more diverse and inclusive scientific community.

Online education has become a common mode for delivering undergraduate science courses to students at scale. Prior to 2020, institutions of higher education increasingly adopted online courses, including science courses [30–32]. In fact, the proportion of undergraduate students taking online courses quadrupled between 2000 and 2012 [33]. In 2020, the COVID-19 pandemic further accelerated the adoption of online science education by colleges and universities. The transition to online learning impacted students around the world [34–37]. In fall 2020, 75% of U.S. institutions delivered courses either completely or partially online [38] and as of fall 2021 many U.S. institutions were continuing some degree of online coursework [39]. Notably, over 500 U.S. universities now offer solely online bachelor’s degrees, many of which are within in the sciences [40].

Despite the ubiquity of online science courses and the high percentage of students struggling with depression, there is a lack of research examining how online science learning environments affect depression. As such, our research group recently conducted an in-depth exploratory interview study with 24 undergraduates with depression who were enrolled in online college science courses at an R1 institution [29]. The interviews probed what specific aspects of online education exacerbate student depression, and what aspects, if any, alleviate students’ depressive symptoms. The self-paced nature of online courses, not needing to show up to class in-person, struggling to develop relationships with other students and the instructor, and struggling to have questions answered online exacerbated student depression. Conversely, the flexibility of coursework and being able to remain anonymous in class helped alleviate students’ depression. The design of this study limited researcher bias by allowing aspects of online science courses that affected depression to emerge from student interviews, as opposed to giving students a list of aspects of online courses that affect their depression from which they could select [41]. However, the small sample size prevented our research group from assessing how common depression is among undergraduate science students and whether particular aspects of online courses are disproportionately detrimental for students with depression who identify within particular demographic groups.

### Current study

Previous studies have not examined the impact of student depression on online learning or whether there are demographic differences in the challenges that students face in online learning environments. To address these gaps in the literature and to assess how online science courses affect student depression at scale, we built upon the interview study by conducting a large-scale survey study to answer the following research questions:

1. To what extent do students in online college science courses report having depression?
2. To what extent does the severity of depression vary among undergraduates in online science courses?
3. What aspects of online science courses exacerbate students’ depression?
4. What aspects of online science courses alleviate students’ depression?

For each research question, we examined whether student demographics, including gender, race/ethnicity, LGBTQ+ status, college generation status, being financially stable, major, division in school, and GPA, predicted the respective outcome.

## Methods

This study was conducted with an approved Institutional Review Board protocol (#13434) from Arizona State University.

To answer the research questions, we surveyed undergraduates enrolled in an in-person degree program who were completing science courses online due to the COVID-19 pandemic at a large research-intensive (R1) institution in the southwest United States where the majority of science courses for the in-person program were offered exclusively online in summer 2020, fall 2020, and spring 2021. We developed a survey based on the exploratory interview study by Mohammed and colleagues that identified aspects of online college science courses that exacerbate and alleviate depressive symptoms among undergraduates [29]. The majority of questions on the survey had been piloted during the preliminary interview study, and it was determined that students were interpreting the questions as intended [42]. After the survey was created, 13 undergraduate and graduate researchers reviewed the survey and suggested modifications using the same criteria to assess all questions (e.g., Is this question grammatically correct? Is the meaning of this question clear?) [43] and the survey was revised accordingly. A copy of the survey questions analyzed for this study is available in the S1 Appendix.

We emailed all instructors (n = 127) teaching life sciences courses at the research-intensive institution and asked if they would be willing to distribute our survey about student mental health to students in their online science courses via the online platform Qualtrics. Thirty-eight (29.9%) instructors agreed to send the survey to the students in their course(s) in exchange for extra credit or for the student to enter a drawing to win one of two $100 gift cards. We recruited from the life sciences department to maximize the number of participants in the study and the number of course experiences students could draw from; the life sciences department is the largest among the natural sciences at the institution and students in these courses were likely enrolled in multiple online science courses at once.

### Screening questions and demographics

The survey asked students how many online college science courses they had enrolled in, which was defined for them as courses in biology, chemistry, geoscience, or physics. Students were then asked if they identify as currently or having previously struggled with depression or a depressive disorder, having never struggled with depression, or prefer not to say. We did not require students to have a formal diagnosis to identify as struggling with depression or a depressive disorder in effort to reduce bias in our study, since mental healthcare is disproportionately unavailable to Black and Latinx individuals, as well as those from low socioeconomic backgrounds [44–46]. Only students who identified as struggling with depression or a depressive disorder were included in the analyses specific to students with depression. Below we review the survey questions and how each was analyzed as it relates to our respective research questions.

### Analyses performed

For each research question, we assessed whether student demographics predicted their responses to specific questions using logistic regression analyses. All analyses were conducted in R [47]. Our predictors included gender (man/woman), race/ethnicity (white, Asian, Black, Latinx), LGBTQ+ status (yes/no), college generation status (first-generation/continuing generation), financially stable (yes/no), STEM major (yes/no), division (upper/lower), and GPA (self-reported on a 4.0 scale). Each predictor variable was determined based on literature suggesting that each may be associated with mental health [4, 13, 23–25, 48, 49]. The reasoning for how each variable was grouped is provided in the S1 Appendix. For all models, we confirmed there were no outliers and checked for multicollinearity among the predictors by assessing the variance inflation factor (VIF) values using the *car* package in R [50, 51]. The VIF values indicated there was no issue with multicollinearity.

#### RQ 1: To what extent do students in online college science courses report having depression?

To investigate the extent to which students in online college science courses report having depression, we calculated the percent of students who reported struggling with depression or a depressive disorder. Then, we used binomial logistic regression to determine to what extent students’ demographics predict whether they report having depression (Model: currently or previously struggled with depression (yes/no) ~ gender + race/ethnicity + LGBTQ+ status + college generation status + financially stable + STEM major + division + GPA).

#### RQ 2: What is the range in severity of depression among undergraduates in online science courses?

Students who identified as struggling with depression were asked to rate the severity of their depression, on average, in the context of online college science courses on a Likert scale including (1) little to no depression, (2) mild depression, (3) moderate depression, and (4) severe depression. We calculated the percent of students who reported each level of depression severity. Using multinomial logistic regression, we assessed to what extent students’ demographics predicted the severity of their depression. Students who identified has having little to no and mild depression were combined into one category to limit our dependent variable to a total of three distinct options because we were most interested in whether there were demographic differences among students with mild depression (little to no or mild), moderate depression, and severe depression (Model: severity of depression (mild/moderate/severe) ~ gender + race/ethnicity + LGBTQ+ status + college generation status + financially stable + STEM major + division + GPA).

#### RQ 3 & 4: What aspects of online science courses exacerbate and alleviate student depression?

In order to investigate the specific aspects of online college science courses that affect student depression, students selected from a list of 15 aspects of online college science courses that could exacerbate their depression (e.g., deciding the pace at which I work through an online science course, struggling to have questions answered) and a list of 11 aspects of online college science courses that could alleviate their depression (e.g., easily getting to know other students in class, being able to engage in an online science course without having to be seen). These aspects of online college science courses that affect depression were developed based on the in-depth interview study of 24 undergraduates examining aspects of online science courses that affect depression [29]. Each of these aspects were organized into one of the following categories: (1) relationship building, (2) getting help or performing well, (3) fear of negative evaluation, or (4) flexible structure. A description of each category and the respective aspects it encompasses are included in the S1 Appendix. We calculated the percentage of students who selected each of the aspects of an online science course that exacerbated their feelings of depression and each of the aspects that alleviated their depression and tested whether there were demographic differences in which students selected each of the aspects using binomial logistic regressions (Model: selected aspect (yes/no) ~ gender + race/ethnicity + LGBTQ+ status + college generation status + financially stable + STEM major + division + GPA).

#### Interpretation of analyses

For analyses of RQ 3 & 4, we controlled for multiple hypothesis testing using a Benjamini-Hochberg (BH) p-value correction with the p.adjust function in the *stats* package in R [52, 53]. We considered all aspects with a BH-adjusted *p* ≤ .05 to be significant. Whether the result of a statistical test is significant or not is continuous rather than dichotomous based on the p-value [54], but we report the results based on the standard of *p* ≤ .05 for simplicity. We acknowledge that p-values greater than .05 can be scientifically meaningful, so we report out all results of statistical tests in the S1 Appendix for the reader’s further interpretation.

## Results

A total of 2,175 students completed the survey and were primarily women (66.9%), white (48.4%), continuing-generation college students (58.8%), and lower division (58.1%). Forty-one percent of students reported they did not consider themselves financially stable at least part of the time and 16.8% identified as a member of the LGBTQ+ community. Student demographics are summarized in Table 1.

**Table 1:** Summary of demographics of survey participants.

Student demographic	Participants % (n)	Student demographic	Participants % (n)
	N = 2175		N = 2175
**Gender**		**College generation status**	
Woman	66.9 (1456)	Continuing-generation	58.8 (1278)
Man	30.7 (667)	First-generation	39.0 (848)
Non-binary	1.3 (28)	Decline to state	2.3 (49)
Other	0.2 (5)	**Financially stable** ^a^	
Decline to state	0.9 (19)	Yes	55.8 (1213)
**Race/ethnicity**		Yes, but only sometimes	28.6 (621)
White	48.4 (1053)	No	12.3 (268)
Hispanic, Latinx, or Spanish Origin	21.6 (469)	Decline to state	3.4 (73)
Asian	15.6 (340)	**Major**	
Other, including multiracial	4.7 (103)	A STEM major	81.2 (1767)
Black or African American	4.5 (97)	A non-STEM major	18.7 (406)
American Indian or Alaska Native	1.8 (39)	Decline to state	0.1 (2)
Pacific Islander	0.7 (15)	**Year in college**	
Decline to state	2.7 (59)	2^nd^ year or less (lower division)	58.1 (1263)
**LGBTQ+**		3^rd^ year or more (upper division)	41.0 (891)
No	78.1 (1698)	Decline to state	1.0 (21)
Yes	16.8 (366)	**GPA**	
Decline to state	5.1 (111)	Mean ± standard deviation	3.5 ± 0.5
		(Range)	(1.3–4.0)

^a^Students were asked whether they considered themselves financially stable (e.g., had enough money for necessity such as groceries and rent) during the time that they have been enrolled in online college science courses.

### Finding 1: Most science undergraduates reported having depression with white students, LGBTQ+ students, women, students who are financially unstable, and students with a lower GPA being most likely to identify as having depression

**Fig 1:**
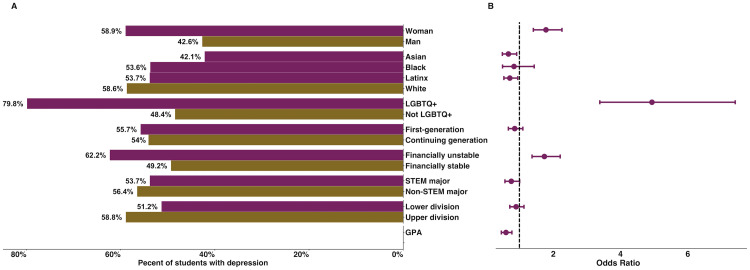
Prevalence of depression and demographic predictors of reporting depression. (A) Percent of each demographic group who reported having depression. (B) Demographic differences in who is more likely to have depression. The vertical dashed line at x = 1 indicates that the group of interest and reference group had equal odds of having depression; error bars that do not cross that vertical dashed line are statistically significant. Reference groups are in brown and include: man, white, non-LGBTQ+, continuing generation, financially stable, non-STEM major, and upper division.

Of the 2,175 participants, 54.2% (n = 1,179) reported experiencing depression. White students were more likely to report depression than Asian and Latinx students; women and LGBTQ+ students were more likely to report depression than men and non-LGBTQ+ students, respectively. Students who were financially unstable were more likely to report depression than students who were financially stable, and the lower a student’s GPA, the more likely they were to report depression. The demographic differences and respective odds ratios are summarized in Fig 1. The full result of the logistic regression and the percentage of students with depression who identify within each demographic category are reported in the S1 Appendix.

### Finding 2: Women, LGBTQ+ students, continuing-generation students, financially unstable students, STEM majors, and lower division students report experiencing more severe depression

**Fig 2:**
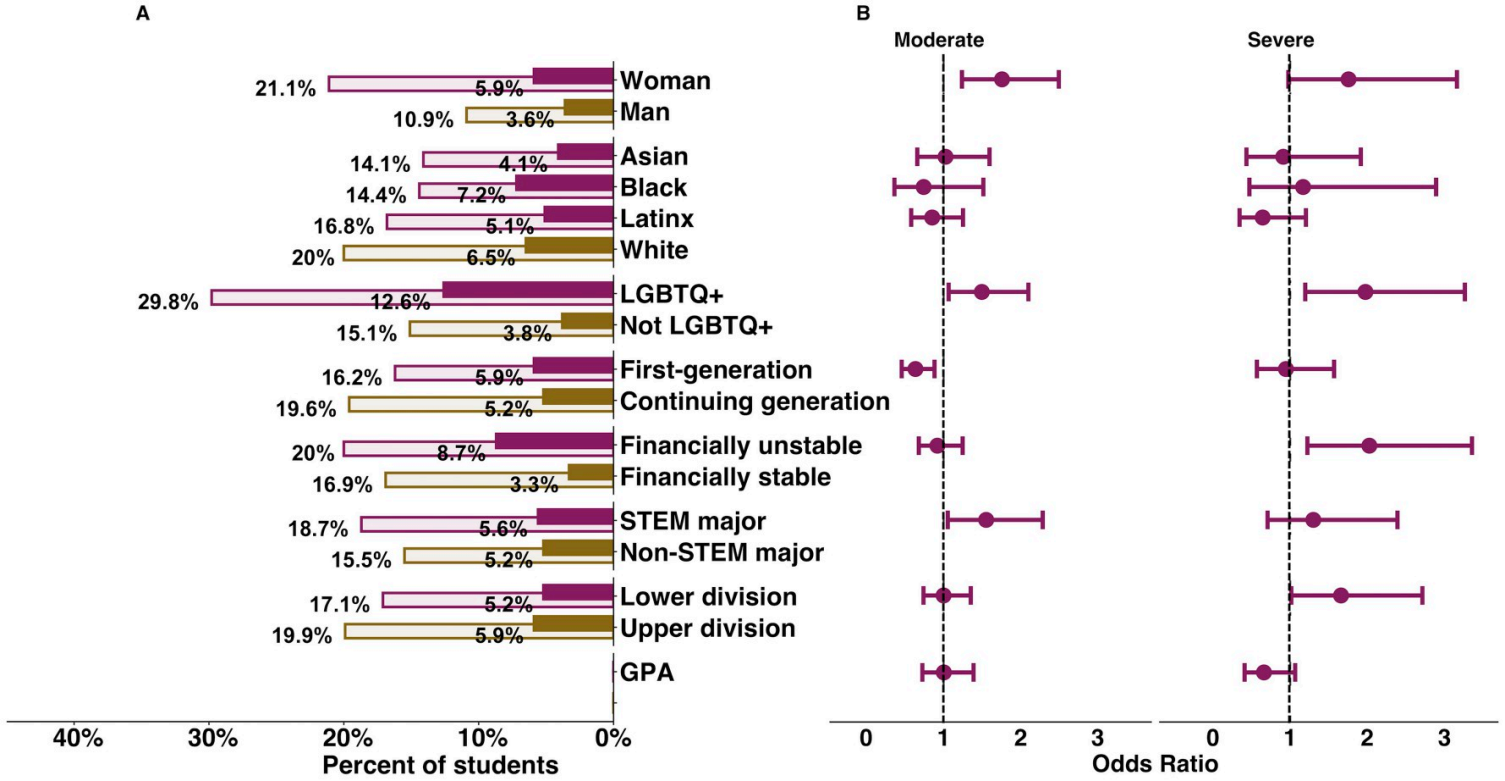
Demographics that predict severity of undergraduate depression. (A) Percent of each demographic group that reported experiencing moderate (lightly shaded bars) or severe (fully shaded bars) depression. (B) Demographic differences in who is more likely to report moderate or severe depression. The vertical dashed line at x = 1 indicates that the group of interest and reference group had equal odds of experiencing moderate or severe depression; error bars that do not cross that vertical dashed line are statistically significant. Reference groups are in brown and include: man, white, non-LGBTQ+, continuing generation, financially stable, non-STEM major, and upper division.

Of the 1,179 students who reported having depression, 56.4% (n = 665) described their depression as little or mild in the context of online college science courses, 33.4% (n = 394) described it as moderate, and 10.2% (n = 120) described it as severe. Women, continuing-generation students, and STEM majors were more likely to report moderate depression than little/mild depression compared to men, first-generation college students, and non-STEM majors, respectively. LGBTQ+ students were more likely to report moderate and severe depression than little or mild depression compared to non-LGBTQ+ students. Students who are financially unstable and lower division students were more likely to report severe depression than little or mild depression compared to financially stable students and upper division students, respectively. The percentage of each demographic group who experienced moderate or severe depression and the respective odds ratios of reporting moderate or severe depression are reported in Fig 2. The full result of the multinomial regression is reported in the S1 Appendix.

### Finding 3: Undergraduates frequently reported that challenges building relationships in the course and aspects related to performing well were aspects of their online college science courses that exacerbated their depression

Students most commonly selected that difficulty getting to know other students in class (61.9%), online monitored proctored testing (57.7%), at-home distractions that interfere with online science courses (54.5%), and difficulty getting to know instructors (51.5%) were aspects of online college science courses that exacerbated their depression. We identified a number of demographic differences with regard to who was more likely to select particular aspects of online college science courses that exacerbated students’ depression. White students were more likely to select an array of aspects of online courses that exacerbated their depression compared to Black students. Women were more likely than men to report that aspects of online courses related to getting help or performing well exacerbated their depression. All significant findings are reported in Table 2 (full results in the S1 Appendix).

**Table 2:** Aspects of online science courses that exacerbate student depression and demographic differences among who selected each aspect.

Aspect of online college science courses that exacerbates depression	% (n) of students who selected the aspect (N = 1,176)^a^	Demographic group who was more likely to select the aspect
Difficulty getting to know other students in class	61.9 (728)	Continuing generation Lower division
Online monitored proctored testing	57.7 (678)	Women White (compared to Black)
At-home distractions that can interfere with online science courses	54.5 (641)	
Difficulty getting to know instructors	51.4 (605)	White (compared to Black)
Difficulty getting help from other students in class	49.7 (584)	White (compared to Black)
Struggling to have questions answered	48.6 (571)	Women White (compared to Black) Financially unstable
Difficulty getting help from instructors	47.5 (559)	White (compared to Black) Lower division
Struggling to communicate effectively with the instructor	46.9 (551)	White (compared to Black)
Comparing myself to other students	46.0 (541)	Women White (compared to Black)
Needing to navigate technology in high-pressure situations (e.g., during online exams)	45.4 (534)	Women Financially unstable

Logistic regression analyses assessed the relationship between whether a student selected a particular aspect of the course that exacerbated their depression and their gender, race/ethnicity, college generation status, LGBTQ+ status, being financially stable, major, GPA, and division in school. Colors represent the broader category of each aspect: yellow = relationship building; purple = getting help or performing well; blue = fear of negative evaluation.

^a^Of the 1,179 students who identified as having depression, three students did not answer the question regarding the aspects of online college science courses which exacerbate their depression, so the percentages reported are out of the 1,176 students who answered the question.

### Finding 4: Undergraduates most commonly reported the flexible course structure and having an instructor who appears to care about mental health as aspects of their online college science courses that helped alleviate their depression

Students most commonly selected that the flexibility of doing coursework *when* they want (65.3%), the flexibility of doing coursework *where* they want (64.2%), having an instructor who appears to care about mental health (58.4%), and being able to engage in an online science course without having to be seen (48.0%) helped alleviate their depression. Women, compared to men, were consistently more likely to select particular aspects of online college science courses that alleviated their depression. All significant findings are reported in Table 3 (full results in the S1 Appendix).

**Table 3:** Aspects of online science courses that alleviate student depression and demographic differences among who selected each aspect.

Aspects of online college science courses that alleviate depression	% (n) of students who selected the aspect (N = 1,175)^a^	Demographic group who was more likely to select each aspect
The flexibility of doing coursework when I want	65.3 (767)	
The flexibility of doing coursework where I want	64.2 (754)	
Having an instructor who appears to care about mental health	58.4 (686)	Women
Being able to engage in an online science course without having to be seen	48.0 (564)	Women
Being anonymous or being able to share my opinion without it being associated with my face	45.4 (534)	Women
Clear communication with instructors	37.5 (441)	Women
Getting questions answered	33.8 (397)	Women
Easily getting help from instructors	31.3 (368)	Women
Easily getting help from other students in class	24.8 (291)	Women
Easily getting to know other students in class	24.8 (291)	

Logistic regression analyses assessed the relationship between whether a student selected a particular aspect of the course that alleviated their depression and their gender, race/ethnicity, college generation status, LGBTQ+ status, being financially stable, major, GPA, and division in school. Colors represent the broader category of each aspect: yellow = relationship building; purple = getting help or performing well; blue = fear of negative evaluation; orange = flexible structure.

^a^Of the 1,179 students who identified as having depression, four students did not answer the question regarding the aspects of online college science courses that alleviate their depression, so the percentages reported are out of the 1,175 students who answered the question.

## Discussion

Depression is likely on the rise among college students [55] and indeed, we found that over half of college science students have experienced depression. The COVID-19 pandemic [56–58] as well as national racial unrest and the increase of racially driven hate crimes in the United States occurring in 2020 and 2021 [59] undoubtedly contributed to the uniquely high rates of depression among science students during the spring 2021 term. However, this research highlights that while depression is common among science students, it disproportionately affects specific groups of science students including LGBTQ+ students, women, financially unstable students, and students with lower GPAs. It is important to note that while white students were more likely to report depression than their Asian and Latinx peers, it is thought that cultural stigmas surrounding depression [60–63] and inadequate access to mental healthcare [44, 45, 64] may influence the underreporting of depression among Asian and Latinx individuals. Additionally, women, LGBTQ+ students, continuing-generation students, financially unstable students, STEM majors, and lower division students experienced more severe depression during the spring 2021 term. Depression is thought to negatively impact student learning [4, 5, 65, 66], likely because it can interfere with one’s cognitive domains [29]. Therefore, the disproportionately high rates and severity of depression experienced among underserved groups in science may be partially contributing to underperformance and attrition in the sciences [67–71].

While curbing the effects of familial and societal events on students’ depression is often beyond the ability of colleges and universities, there is increasing evidence that several aspects of college itself may have an impact on student mental health. This study adds to the recent research establishing that science learning environments in particular may affect depression [13, 14, 72]. Our foundational interview study [29] provided evidence that aspects of online learning related to social interactions, getting help or performing well, being evaluated by others, and flexibility affect student depression and the data presented in this study imply that these findings can be generalized to a larger population of students.

The social aspects of online learning greatly affect students’ depression. Not getting to know other students in class was the most common aspect of online courses that students selected as exacerbating their depression and not getting to know instructors was reported by over half of participants. The lack of opportunities for both formal and informal interactions in online courses [73, 74] makes it difficult to form student-student and student-instructor relationships, which likely results in or amplifies feelings of loneliness, a common symptom of depression [13, 15, 29]. Further, the lack of student-student interactions may be particularly detrimental for students’ depression in spring 2021, given that the COVID-19 pandemic resulted in social isolation for most individuals [75–78].

In addition to the difficulty of developing relationships online, online proctored testing and at-home distractions that interfered with students’ courses were also selected by over half of students as aspects that exacerbated their depression. Proctored online testing has been found to exacerbate student anxiety [79–83] and to create challenges for students with disabilities [84, 85]. The threat of being “flagged” as cheating when carrying out routine behaviors such as looking down or even needing to step away for health reasons has been described to take a profound toll on students’ mental health, which is echoed by students in this study. Additionally, at home distractions have been shown to increase anxiety levels of students in online courses because such distractions make it harder for students to focus and concentrate [82, 86], which may also lead to increased feelings of frustration and hopelessness related to depression.

Both aspects of flexible online instruction that were listed for students, completing coursework where students want and completing coursework when students want, emerged as the top two aspects that helped alleviate student depression. Since depressive episodes can result in fatigue as well as diminished abilities to think or concentrate, having the flexibility to engage in coursework when a student feels best likely prevents the exacerbation of depressive symptoms, such as feelings of guilt associated with not performing well in class [2, 29]. Our previous study found that not having to physically go to class can be detrimental for students with depression because it can discourage them from completing activities of daily living such as brushing one’s teeth or hair or getting dressed, but students also described that having to be physically present when experiencing a major depressive episode or losing points for being physically absent from class took a toll on their depression [29]. As such, it was difficult to discern from the interview study what the overall effect of needing to show up to class in person might have on students with depression, but this quantitative study suggests that students may see being able to complete coursework from where they want as more of an advantage for their depression than a disadvantage.

Overall, we found few notable trends with regard to the demographic differences in what aspects of online college science courses alleviated and exacerbated undergraduates’ depression. White students were more likely than Black students to report that aspects of their courses affected their depression. However, given the state of racial unrest of the United States before and during the spring 2021 term, the state of the nation and racial injustices may have been much more impactful on Black students’ depression than aspects of online learning [87–90]. Women were more likely than men to report that an array of aspects of online college science courses exacerbated and helped alleviate their depression. This may be because women often experience stereotype threat, defined as having the potential to confirm a negative stereotype about one’s group [91], in science disciplines which may threaten their mental health [92]. Additionally, women are more likely to seek help for their mental health, so may be better able to identify aspects of online courses that exacerbate or alleviate their depression [93–95].

As we strive for an inclusive science learning environment for everyone, we propose recommendations based on the data presented in this study to help instructors create more inclusive online science courses for students with depression. Students benefit from building relationships online with both peers and instructors [96–100] and our data suggest this is likely protective of students’ mental health. As such, integrating opportunities for students to interact with each other (e.g., breakout rooms, small group work, discussion board posts) and the instructor (e.g., holding virtual office hours) may be effective in helping to build such relationships and improve mental health. Additionally, monitored proctored testing was frequently reported as an aspect that negatively affected students’ depression and has also been found to exacerbate student anxiety [82]. While we acknowledge that the elimination of proctoring software may lead to an increase in academic dishonesty, we encourage instructors to consider alternative forms of evaluation. For example, replacing infrequent high-stakes testing with more frequent low-stakes evaluation may disproportionately benefit students’ mental health and consequently students with underrepresented and underserved identities [71, 101–103]. Finally, students reported that it helps to alleviate their depression when they perceive their instructor cares about mental health. Making an announcement at the beginning of the term acknowledging the importance of mental health, providing students with resources such as information about the disability resource center, and putting a clause in the syllabus about the extent to which one values student mental health [104] are all simple steps that have the potential to positively impact student mental health.

### Limitations and future directions

This study was conducted during the COVID-19 pandemic. While we asked students to report on aspects of online courses that affect their depression that were unrelated to the pandemic, it is likely that the stress and isolation associated with COVID-19 increased the number of students who identified as having depression. Further, this study was conducted at a large R1 institution in the U.S., so the results may not be generalizable across other institution types or other countries. Students self-identified as having depression and were not required to have a diagnosis of depression to take part in this study because mental healthcare is disproportionately available to more privileged groups [44–46]. However, self-report of depression has been found to be relatively accurate and appropriate in non-clinical contexts [105]. While this study identified aspects of online college science courses that students reported alleviated their depressive symptoms, future research could experimentally assess to what extent a change in a course (e.g., increasing flexibility, facilitating student-student interactions) results in a decrease of depressive symptoms among students. This would allow for more concrete recommendations about how to create more inclusive science courses for students with depression.

## Supporting information

S1 Appendix Supplementary material. All supplementary material for the manuscript including a copy of the survey and full results of regression analyses.
(DOCX)

S1 Dataset De-identified dataset. Dataset used in all data analyses.
(CSV)
