# Supplementary material for: Aspects of online college science courses that alleviate and exacerbate undergraduate depression
Source: PLoS One. 2022 Jun 1;17(6):e0269201. doi: 10.1371/journal.pone.0269201 (PMC9159593; doi:10.1371/journal.pone.0269201)
Supplement: S1 Appendix — All supplementary material for the manuscript including a copy of the survey and full results of regression analyses. (DOCX) [file pone.0269201.s001.docx]

**S1 Appendix for**

***Aspects of online college science courses that alleviate and exacerbate undergraduate depression***

**Authors:** Carly A. Busch^1^, Tasneem F. Mohammed^1^, Erika M. Nadile^1^, Katelyn M. Cooper^1^^

^1^Research for Inclusive STEM Education Center, School of Life Sciences, Arizona State University

^Corresponding author

**This supplement document contains the following:**

| **Item** | **Page number** |
| --- | --- |
| Copy of survey questions analyzed | 2 – 5 |
| Literature-supported justification for the grouping of the variables in the regression models | 6 |
| Categorization of the aspects of online college science courses that exacerbate or alleviate students’ depression (Table S1) | 7 – 8 |
| Demographic summary of students with depression in the study (Table S2) | 9 |
| Summary of full result of the logistic regression to identify demographic differences in who reports having depression (Table S3) | 10 |
| Summary of full result of the multinomial regression to identify demographic differences in the severity of student depression (Table S4) | 11 |
| Summary of full result of the logistic regressions to identify demographic differences in the aspects of online college science courses that exacerbate student depression (Table S5) | 12 – 17 |
| Summary of full result of the logistic regressions to identify demographic differences in the aspects of online college science courses that alleviate student depression (Table S6) | 18 – 21 |

**Copy of survey questions analyzed**

Please answer the following questions about the **SCIENCE** courses that you have completed at Arizona State University.

Q152 We are interested in creating more inclusive environments for students with depression at ASU. For this reason, we will be asking you a set of questions about your experience with depression in online courses. Please feel free to skip any question you do not want to answer.

Q132 Please indicate how you most closely identify. You do not need to have a formal diagnosis to identify as having currently or previously struggled with depression or a depressive disorder.

o Currently or having previously struggled with depression or a depressive disorder

o Having never struggled with depression

o Prefer not to say

Q154 Have you been diagnosed with **depression**?

o Yes

o No

o Prefer not to say

Q155 Are you currently being treated, or have you previously been treated for **depression**? Select all that apply.

▢        Medication

▢        Counseling/therapy (e.g., working with a psychologist or therapist)

▢        Other, please describe  ________________________________________________

▢        I am not/have not been treated for depression

▢        Prefer not to say

Q103 How would you rate the severity of your depression, on average, in the context of **ONLINE college science courses**?

o Little to no depression

o Mild depression

o Moderate depression

o Severe depression

Q104 Please select all aspects of **ONLINE college science courses** that might make your feelings of depression **worse**.

▢        Difficulty getting to know other students in class

▢        Difficulty getting help from other students in class

▢        Needing to talk with students who I don’t know during online group work

▢        Difficulty getting to know instructors

▢        Difficulty getting help from instructors

▢        Comparing myself to other students

▢        Not having to show up in person to online science courses

▢        Deciding the pace at which I work through an online science course

▢        Being on camera

▢        Needing to navigate technology in high-pressure situations (e.g., during exams)

▢        Online monitored proctored testing

▢        Struggling to have questions answered

▢        Struggling to communicate effectively with the instructor

▢        At-home distractions that can interfere with online science courses

▢        The potential for personal technology issues (e.g., unstable internet connection)

▢        Other, please describe  ________________________________________________

▢        Nothing related to online courses makes my feelings of depression worse

Q105 Please select all aspects of **ONLINE college science courses** that might **help you manage** your depression.

▢        Easily getting to know other students in class

▢        Easily getting help from other students in class

▢        Easily getting to know instructors

▢        Easily getting help from instructors

▢        Having an instructor who appears to care about mental health

▢        The flexibility of doing coursework *when* I want

▢        The flexibility of doing coursework *where* I want

▢        Being able to engage in an online science course without having to be seen

▢        Getting questions answered

▢        Clear communication with instructors

▢        Being anonymous or being able to share my opinion without it being associated with my face

▢        Other, please describe  ________________________________________________

▢        Nothing related to online courses helps me manage my depression

Q50 I most closely identify as

o Woman

o Man

o Non-binary

o Other, please describe  ________________________________________________

o Prefer not to say

Q54 I most closely identify as

o American Indian or Alaska Native

o Asian

o Black or African American

o Hispanic, Latinx, or Spanish origin

o Pacific Islander

o White/Caucasian

o Other, please describe  ________________________________________________

o Prefer not to say

Q52 What is your parent or guardian's highest level of education? If you have more than one parent or guardian with differing levels of education, choose the higher of the two.

o Less than high school completed

o High school diploma or GED

o Some college but no degree

o Associate degree (e.g., AA, AS)

o Bachelor's degree (e.g., BA, AB, BS)

o Master's degree (e.g., MA, MS, MEng, MEd, MSW, MBA)

o Higher than a master's degree (e.g., PhD, MD, JD)

o Prefer not to say

Q114 During the time you have taken online science courses, have you considered yourself financially stable (e.g., had enough money for necessities such as groceries and rent)?

o Yes

o Yes, but only sometimes

o No

o Prefer not to say

Q77 What is your GPA (on a 4.0 scale)?

Q62 How long have you attended college while pursuing your undergraduate degree?

o 1 year or less

o 2 years

o 3 years

o 4 years

o 5 years or more

o I have graduated with my undergraduate degree

o Prefer not to say

Q64 Do you identify as a member of the LGBTQIA* community?

*lesbian, gay, bisexual, transgender, queer/questioning, intersex, asexual/aromantic

o Yes

o No

o Prefer not to say

**If yes:** Please select the word or words that best describe your identity:

▢        Lesbian

▢        Gay

▢        Bisexual

▢        Queer

▢        Transgender

▢        Intersex

▢        Asexual

▢        Other, please describe  ________________________________________________

▢        Prefer not to say

Q64 Please select the major that is closest to yours. If you have more than one major, please choose all that apply.

▢        Biology

▢        Biochemistry

▢        Chemistry

▢        Physics

▢        Geosciences

▢        Business

▢        English

▢        Psychology

▢        Sociology

Other, please describe  ________________________________________________

**Literature-supported justification for the grouping of the variables in the regression models**

We recognize that not all individuals identify as gender binary (man or woman) (Cooper et al. 2020), but there were too few students who identified as a gender other than man or woman in our sample to create a third category for analyses. Additionally, we excluded students who identified as American Indian or Alaska Native, Pacific Islander, multiracial, or as a race not listed on the survey from analyses owing to low sample sizes. We grouped students into science, technology, engineering, and math (STEM) majors and non-STEM majors because we perceived that students who are pursuing STEM majors may have different experiences in online college science courses from students pursuing non-STEM majors (Cotner et al. 2017, Michaluk et al. 2018). We grouped students in their first or second year of undergraduate into the single category “lower division” and students in their third or later year into “upper division.”

References:

Cooper, Katelyn M., Anna Jo J. Auerbach, Jordan D. Bader, Amy S. Beadles-Bohling, Jacqueline A. Brashears, Erica Cline, Sarah L. Eddy, Deanna B. Elliott, Elijah Farley, and Linda Fuselier. 2020. “Fourteen Recommendations to Create a More Inclusive Environment for LGBTQ+ Individuals in Academic Biology.” *CBE—Life Sciences Education* 19 (3): es6. https://doi.org/10/gg5wxf.

Cotner, Sehoya, Seth Thompson, and Robin Wright. 2017. “Do Biology Majors Really Differ from Non–STEM Majors?” *CBE—Life Sciences Education* 16 (3): ar48.

Michaluk, Lynnette, Rachel Stoiko, Gay Stewart, and John Stewart. 2018. “Beliefs and Attitudes about Science and Mathematics in Pre-Service Elementary Teachers, STEM, and Non-STEM Majors in Undergraduate Physics Courses.” *Journal of Science Education and Technology* 27 (2): 99–113.

**Table S1.** Categorization of the aspects of online college science courses that exacerbate or alleviate student depression.

| **Category** | | **Description** | **Aspect that exacerbates depression** | **Aspect that alleviates depression** |
| --- | --- | --- | --- | --- |
|  | Relationship building | These aspects of online courses are related to students’ ability to form relationships with their peers or the instructor. | Difficulty getting to know other students in class | Having an instructor who appears to care about mental health |
|  |  |  | Difficulty getting to know instructors | Easily getting to know other students in class |
|  |  |  | Struggling to communicate effectively with the instructor | Easily getting to know instructors |
|  | Getting help or performing well | These aspects of online courses are related to students’ ability to access course material, content mastery, and completing graded assignments such as exams. | Online monitored proctored testing | Clear communication with instructors |
|  |  |  | At-home distractions that can interfere with online science courses | Getting questions answered |
|  |  |  | Difficulty getting help from other students in class | Easily getting help from instructors |
|  |  |  | Struggling to have questions answered | Easily getting help from other students in class |
|  |  |  | Difficulty getting help from instructors |  |
|  |  |  | Needing to navigate technology in high-pressure situations (e.g., during exams) |  |
|  |  |  | The potential for personal technology issues (e.g., unstable internet connection) |  |
|  | Fear of negative evaluation | These aspects of online courses are related to students’ fear of negative evaluation from others when participating in class or being judged by their peers or the instructor. | Comparing myself to others | Being able to engage in an online science course without having to be seen |
|  |  |  | Needing to talk to students who I don’t know during online group work | Being anonymous or being able to share my opinion without it being associated with my face |
|  |  |  | Being on camera |  |
|  | Flexible structure | These aspects of online courses are related to the flexible schedule often associated with online coursework. | Not having to show up in person to online science courses | The flexibility of doing coursework when I want |
|  |  |  | Deciding the pace at which I work through an online science course | The flexibility of doing coursework where I want |

**Table S2.** Demographic summary of students with depression in the study.

| **Student demographic** | | **Participants % (n)**  **N = 1,179** | **Student demographic** | | **Participants % (n)**  **N = 1,179** |
| --- | --- | --- | --- | --- | --- |
| **Gender** | |  | **College generation status** | |  |
|  | Woman | 72.7 (857) |  | Continuing-generation | 58.5 (690) |
|  | Man | 24.1 (284) |  | First-generation | 40.0 (472) |
|  | Non-binary | 2.0 (24) |  | Decline to state | 1.4 (17) |
|  | Other | 0.4 (5) | **Financially stable^a^** | |  |
|  | Decline to state | 0.8 (9) |  | Yes | 50.6 (597) |
| **Race/ethnicity** | |  |  | Yes, but only sometimes | 32.7 (386) |
|  | White | 52.3 (617) |  | No | 14.2 (167) |
|  | Hispanic, Latinx, or Spanish Origin | 21.4 (252) |  | Decline to state | 2.5 (29) |
|  | Asian | 12.1 (143) | **Major** | |  |
|  | Other, including multiracial | 5.3 (63) |  | A STEM major | 80.5 (949) |
|  | Black or African American | 4.4 (52) |  | A non-STEM major | 19.4 (229) |
|  | American Indian or Alaska Native | 2.0 (23) |  | Decline to state | 0.1 (1) |
|  | Pacific Islander | 0.6 (7) | **Year in college** | |  |
|  | Decline to state | 1.9 (22) |  | 2^nd^ year or less (lower division) | 54.9 (647) |
| **LGBTQ+** | |  |  | 3^rd^ year or more (upper division) | 44.4 (524) |
|  | No | 69.6 (821) |  | Decline to state | 0.7 (8) |
|  | Yes | 24.8 (292) | **GPA** | |  |
|  | Decline to state | 5.6 (66) |  | Mean ± standard deviation | 3.5 ± 0.5 |
|  | |  |  | (Range) | (1.5 – 4.0) |
| ^a^Students were asked whether they considered themselves financially stable (e.g., had enough money for necessity such as groceries and rent) during the time that they have been enrolled in online college science courses. | | | | | |

**Table S3.** Summary of logistic regression model of whether students report having depression and their gender, race/ethnicity, LGBTQ+ status, college generation status, financially stable, major, division, and GPA.

| **Variable** | **B** | **SE B** | **β** | **OR** | ***p*** |
| --- | --- | --- | --- | --- | --- |
| Intercept | 2.02 | 0.52 | 0.55 | 7.50 | < .001 |
| Gender (Woman) | 0.58 | 0.12 | 0.27 | 1.78 | < .001 |
| Race/ethnicity (Asian) | -0.41 | 0.16 | -0.15 | 0.66 | .01 |
| Race/ethnicity (Black) | -0.19 | 0.27 | -0.04 | 0.83 | .50 |
| Race/ethnicity (Latinx) | -0.35 | 0.15 | -0.15 | 0.71 | .02 |
| LGBTQ+ (yes) | 1.60 | 0.20 | 0.60 | 4.93 | < .001 |
| College generation status (first-gen) | -0.16 | 0.13 | -0.08 | 0.85 | .21 |
| Not financially stable | 0.55 | 0.12 | 0.27 | 1.73 | < .001 |
| STEM major | -0.29 | 0.15 | -0.11 | 0.75 | .05 |
| Division (lower) | -0.11 | 0.12 | -0.06 | 0.89 | .34 |
| GPA | -0.52 | 0.13 | -0.24 | 0.59 | < .001 |
| B represents unstandardized coefficients, whereas β represents standardized coefficients. OR represents the odds ratio (calculated as e^B^). Focus categories are provided in parentheses in the first column and reference groups are men, white, non-LGBTQ+, continuing generation, financially stable, non-STEM major, and upper division. | | | | | |

**Table S4.** Summary of multinomial regression model of the severity of student depression and their gender, race/ethnicity, LGBTQ+ status, college generation status, financially stable, major, division, and GPA.

| **Variable** | **Moderate** | | | | **Severe** | | | |
| --- | --- | --- | --- | --- | --- | --- | --- | --- |
|  | **B** | **SE B** | **OR** | ***p*** | **B** | **SE B** | **OR** | ***p*** |
| Intercept | -1.21 | 0.66 | 0.30 | .07 | -1.77 | 0.99 | 0.17 | .07 |
| Gender (woman) | 0.56 | 0.18 | 1.76 | .002 | 0.56 | 0.30 | 1.76 | .06 |
| Race/ethnicity (Asian) | 0.03 | 0.23 | 1.03 | .91 | -0.09 | 0.38 | 0.91 | .80 |
| Race/ethnicity (Black) | -0.30 | 0.36 | 0.74 | .41 | 0.15 | 0.46 | 1.17 | .74 |
| Race/ethnicity (Latinx) | -0.16 | 0.20 | 0.85 | .42 | -0.44 | 0.32 | 0.64 | .17 |
| LGBTQ+ (yes) | 0.40 | 0.17 | 1.50 | .02 | 0.68 | 0.26 | 1.97 | .008 |
| College generation status (first-gen) | -0.45 | 0.17 | 0.64 | .007 | -0.06 | 0.26 | 0.94 | .82 |
| Not financially stable | -0.08 | 0.16 | 0.92 | .59 | 0.71 | 0.26 | 2.03 | .006 |
| STEM major | 0.44 | 0.20 | 1.55 | .03 | 0.26 | 0.31 | 1.30 | .40 |
| Division (lower) | 0.00 | 0.15 | 1.00 | .997 | 0.51 | 0.25 | 1.66 | .04 |
| GPA | 0.00 | 0.17 | 1.00 | .98 | -0.42 | 0.24 | 0.66 | .09 |
| B represents unstandardized coefficients. OR represents the odds ratio (calculated as e^B^). Focus categories are provided in parentheses in the first column and reference groups are men, white, non-LGBTQ+, continuing generation, financially stable, non-STEM major, and upper division. | | | | | | | | |

**Table S5.** Summary of full result of the logistic regressions to identify demographic differences in the aspects of online college science courses that exacerbate student depression. B represents unstandardized coefficients, whereas β represents standardized coefficients. OR represents the odds ratio (calculated as e^B^). Adjusted p-value is based on B-H corrections by demographic group across all aspects in the table. Focus categories are provided in the second column and reference groups are men, white, non-LGBTQ+, continuing generation, financially stable, non-STEM major, and upper division.

| **Aspect** | **Variable** | **B** | **SE B** | **β** | **OR** | ***p*** | **Adjusted *p*** |
| --- | --- | --- | --- | --- | --- | --- | --- |
| Being on camera | Intercept | 0.24 | 0.14 | 0.38 | 1.27 | 0.0874 | NA |
|  | Woman | 0.12 | 0.04 | 0.05 | 1.13 | 0.0013 | 0.0073 |
|  | Asian | -0.03 | 0.05 | -0.01 | 0.97 | 0.4914 | 0.7385 |
|  | Black | 0.04 | 0.07 | 0.01 | 1.04 | 0.6142 | 0.6142 |
|  | Latinx | 0.00 | 0.04 | 0.00 | 1.00 | 0.9110 | 0.9110 |
|  | LGBTQ+ | 0.08 | 0.04 | 0.03 | 1.08 | 0.0394 | 0.1718 |
|  | First-gen | 0.01 | 0.04 | 0.01 | 1.01 | 0.7228 | 0.9328 |
|  | Financially unstable | 0.13 | 0.03 | 0.06 | 1.13 | 0.0002 | 0.0029 |
|  | STEM major | 0.08 | 0.04 | 0.03 | 1.09 | 0.0403 | 0.1712 |
|  | Lower division | 0.07 | 0.03 | 0.03 | 1.07 | 0.0371 | 0.1057 |
|  | GPA | -0.04 | 0.03 | -0.02 | 0.96 | 0.2729 | 0.5588 |
| Struggling to communicate effectively with the instructor | Intercept | 0.48 | 0.14 | 0.47 | 1.62 | 0.0009 | NA |
|  | Woman | 0.05 | 0.04 | 0.02 | 1.05 | 0.1853 | 0.2250 |
|  | Asian | -0.07 | 0.05 | -0.02 | 0.93 | 0.1627 | 0.7374 |
|  | Black | -0.23 | 0.08 | -0.05 | 0.79 | 0.0021 | 0.0091 |
|  | Latinx | -0.11 | 0.04 | -0.05 | 0.90 | 0.0105 | 0.1789 |
|  | LGBTQ+ | 0.02 | 0.04 | 0.01 | 1.02 | 0.6536 | 0.8716 |
|  | First-gen | -0.02 | 0.04 | -0.01 | 0.98 | 0.5718 | 0.9328 |
|  | Financially unstable | 0.08 | 0.03 | 0.04 | 1.08 | 0.0254 | 0.0864 |
|  | STEM major | 0.06 | 0.04 | 0.02 | 1.06 | 0.1746 | 0.4241 |
|  | Lower division | 0.07 | 0.03 | 0.04 | 1.08 | 0.0339 | 0.1057 |
|  | GPA | -0.04 | 0.04 | -0.02 | 0.96 | 0.3238 | 0.5588 |
| Comparing myself to other students | Intercept | 0.43 | 0.14 | 0.45 | 1.53 | 0.0030 | NA |
|  | Woman | 0.10 | 0.04 | 0.05 | 1.11 | 0.0059 | 0.0200 |
|  | Asian | 0.02 | 0.05 | 0.01 | 1.02 | 0.6459 | 0.7385 |
|  | Black | -0.27 | 0.07 | -0.06 | 0.76 | 0.0003 | 0.0016 |
|  | Latinx | -0.04 | 0.04 | -0.02 | 0.96 | 0.3336 | 0.8825 |
|  | LGBTQ+ | 0.09 | 0.04 | 0.04 | 1.10 | 0.0155 | 0.1314 |
|  | First-gen | -0.01 | 0.04 | -0.01 | 0.99 | 0.7253 | 0.9328 |
|  | Financially unstable | 0.06 | 0.03 | 0.03 | 1.07 | 0.0597 | 0.1396 |
|  | STEM major | 0.07 | 0.04 | 0.03 | 1.08 | 0.0751 | 0.2555 |
|  | Lower division | 0.03 | 0.03 | 0.02 | 1.03 | 0.3542 | 0.4462 |
|  | GPA | -0.05 | 0.04 | -0.02 | 0.96 | 0.2037 | 0.5588 |
| Difficulty getting help from instructors | Intercept | 0.37 | 0.15 | 0.47 | 1.44 | 0.0122 | NA |
|  | Woman | 0.06 | 0.04 | 0.03 | 1.06 | 0.1168 | 0.1528 |
|  | Asian | -0.03 | 0.05 | -0.01 | 0.98 | 0.6264 | 0.7385 |
|  | Black | -0.21 | 0.08 | -0.05 | 0.81 | 0.0068 | 0.0194 |
|  | Latinx | -0.06 | 0.04 | -0.02 | 0.94 | 0.1851 | 0.7866 |
|  | LGBTQ+ | 0.00 | 0.04 | 0.00 | 1.00 | 0.9999 | 0.9999 |
|  | First-gen | 0.00 | 0.04 | 0.00 | 1.00 | 0.8963 | 0.9328 |
|  | Financially unstable | 0.01 | 0.03 | 0.01 | 1.01 | 0.7124 | 0.7801 |
|  | STEM major | 0.03 | 0.04 | 0.01 | 1.03 | 0.4688 | 0.6047 |
|  | Lower division | 0.10 | 0.03 | 0.05 | 1.11 | 0.0031 | 0.0261 |
|  | GPA | 0.00 | 0.04 | 0.00 | 1.00 | 0.9558 | 0.9558 |
| Difficulty getting help from other students in class | Intercept | 0.28 | 0.14 | 0.49 | 1.33 | 0.0509 | NA |
|  | Woman | 0.06 | 0.04 | 0.03 | 1.07 | 0.0956 | 0.1478 |
|  | Asian | 0.13 | 0.05 | 0.04 | 1.14 | 0.0123 | 0.2089 |
|  | Black | -0.22 | 0.08 | -0.05 | 0.81 | 0.0044 | 0.0149 |
|  | Latinx | -0.03 | 0.04 | -0.01 | 0.97 | 0.4896 | 0.8825 |
|  | LGBTQ+ | -0.01 | 0.04 | 0.00 | 0.99 | 0.8961 | 0.9999 |
|  | First-gen | -0.01 | 0.04 | 0.00 | 0.99 | 0.8627 | 0.9328 |
|  | Financially unstable | 0.04 | 0.03 | 0.02 | 1.04 | 0.2200 | 0.3400 |
|  | STEM major | 0.04 | 0.04 | 0.02 | 1.04 | 0.3109 | 0.5286 |
|  | Lower division | 0.07 | 0.03 | 0.03 | 1.07 | 0.0435 | 0.1057 |
|  | GPA | 0.02 | 0.04 | 0.01 | 1.02 | 0.5763 | 0.8164 |
| Difficulty getting to know instructors | Intercept | 0.22 | 0.15 | 0.52 | 1.24 | 0.1373 | NA |
|  | Woman | 0.06 | 0.04 | 0.03 | 1.07 | 0.0941 | 0.1478 |
|  | Asian | 0.01 | 0.05 | 0.00 | 1.01 | 0.8342 | 0.8342 |
|  | Black | -0.17 | 0.08 | -0.04 | 0.84 | 0.0218 | 0.0457 |
|  | Latinx | -0.07 | 0.04 | -0.03 | 0.93 | 0.0850 | 0.4816 |
|  | LGBTQ+ | -0.02 | 0.04 | -0.01 | 0.98 | 0.6378 | 0.8716 |
|  | First-gen | -0.02 | 0.04 | -0.01 | 0.98 | 0.5550 | 0.9328 |
|  | Financially unstable | 0.01 | 0.03 | 0.01 | 1.01 | 0.7342 | 0.7801 |
|  | STEM major | -0.01 | 0.04 | 0.00 | 0.99 | 0.7699 | 0.7699 |
|  | Lower division | 0.06 | 0.03 | 0.03 | 1.07 | 0.0654 | 0.1389 |
|  | GPA | 0.07 | 0.04 | 0.04 | 1.08 | 0.0400 | 0.2264 |
| Difficulty getting to know other students in class | Intercept | 0.20 | 0.14 | 0.62 | 1.23 | 0.1356 | NA |
|  | Woman | 0.01 | 0.04 | 0.00 | 1.01 | 0.8203 | 0.8203 |
|  | Asian | 0.11 | 0.05 | 0.04 | 1.11 | 0.0296 | 0.2518 |
|  | Black | -0.15 | 0.07 | -0.03 | 0.86 | 0.0347 | 0.0537 |
|  | Latinx | -0.03 | 0.04 | -0.01 | 0.97 | 0.5250 | 0.8825 |
|  | LGBTQ+ | 0.00 | 0.04 | 0.00 | 1.00 | 0.9707 | 0.9999 |
|  | First-gen | -0.11 | 0.03 | -0.05 | 0.90 | 0.0023 | 0.0390 |
|  | Financially unstable | 0.01 | 0.03 | 0.00 | 1.01 | 0.8763 | 0.8763 |
|  | STEM major | 0.01 | 0.04 | 0.01 | 1.01 | 0.7419 | 0.7699 |
|  | Lower division | 0.19 | 0.03 | 0.09 | 1.20 | 0.0000 | 0.0000 |
|  | GPA | 0.10 | 0.03 | 0.05 | 1.10 | 0.0050 | 0.0852 |
| At-home distractions that can interfere with online science courses | Intercept | 0.23 | 0.14 | 0.54 | 1.26 | 0.1078 | NA |
|  | Woman | 0.08 | 0.04 | 0.03 | 1.08 | 0.0383 | 0.0815 |
|  | Asian | -0.02 | 0.05 | -0.01 | 0.98 | 0.6500 | 0.7385 |
|  | Black | -0.15 | 0.08 | -0.03 | 0.86 | 0.0525 | 0.0744 |
|  | Latinx | 0.02 | 0.04 | 0.01 | 1.02 | 0.6748 | 0.8825 |
|  | LGBTQ+ | 0.11 | 0.04 | 0.05 | 1.11 | 0.0060 | 0.1018 |
|  | First-gen | 0.06 | 0.04 | 0.03 | 1.06 | 0.1252 | 0.6377 |
|  | Financially unstable | 0.06 | 0.03 | 0.03 | 1.06 | 0.0808 | 0.1526 |
|  | STEM major | 0.10 | 0.04 | 0.04 | 1.11 | 0.0129 | 0.1051 |
|  | Lower division | -0.03 | 0.03 | -0.01 | 0.97 | 0.4560 | 0.4845 |
|  | GPA | 0.03 | 0.04 | 0.01 | 1.03 | 0.3928 | 0.6070 |
| Needing to navigate technology in high-pressure situations (e.g., during exams) | Intercept | 0.14 | 0.14 | 0.46 | 1.15 | 0.3377 | NA |
|  | Woman | 0.11 | 0.04 | 0.05 | 1.11 | 0.0057 | 0.0200 |
|  | Asian | -0.05 | 0.05 | -0.02 | 0.95 | 0.2904 | 0.7385 |
|  | Black | -0.13 | 0.08 | -0.03 | 0.87 | 0.0761 | 0.0996 |
|  | Latinx | 0.01 | 0.04 | 0.00 | 1.01 | 0.8494 | 0.9025 |
|  | LGBTQ+ | 0.05 | 0.04 | 0.02 | 1.05 | 0.2097 | 0.5941 |
|  | First-gen | -0.05 | 0.04 | -0.02 | 0.95 | 0.1933 | 0.6525 |
|  | Financially unstable | 0.09 | 0.03 | 0.05 | 1.10 | 0.0070 | 0.0399 |
|  | STEM major | 0.10 | 0.04 | 0.04 | 1.10 | 0.0185 | 0.1051 |
|  | Lower division | -0.03 | 0.03 | -0.01 | 0.97 | 0.3937 | 0.4462 |
|  | GPA | 0.04 | 0.04 | 0.02 | 1.05 | 0.2195 | 0.5588 |
| Not having to show up in person to online science courses | Intercept | 0.11 | 0.13 | 0.31 | 1.11 | 0.4202 | NA |
|  | Woman | 0.06 | 0.04 | 0.02 | 1.06 | 0.1166 | 0.1528 |
|  | Asian | -0.06 | 0.05 | -0.02 | 0.94 | 0.2169 | 0.7374 |
|  | Black | -0.09 | 0.07 | -0.02 | 0.92 | 0.2256 | 0.2740 |
|  | Latinx | -0.03 | 0.04 | -0.01 | 0.98 | 0.5269 | 0.8825 |
|  | LGBTQ+ | 0.07 | 0.04 | 0.03 | 1.07 | 0.0518 | 0.1761 |
|  | First-gen | -0.08 | 0.03 | -0.04 | 0.92 | 0.0145 | 0.1230 |
|  | Financially unstable | 0.04 | 0.03 | 0.02 | 1.04 | 0.2451 | 0.3473 |
|  | STEM major | 0.05 | 0.04 | 0.02 | 1.06 | 0.1621 | 0.4241 |
|  | Lower division | 0.03 | 0.03 | 0.01 | 1.03 | 0.3762 | 0.4462 |
|  | GPA | 0.03 | 0.03 | 0.02 | 1.03 | 0.3287 | 0.5588 |
| Deciding the pace at which I work through an online science course | Intercept | 0.41 | 0.13 | 0.27 | 1.51 | 0.0017 | NA |
|  | Woman | 0.08 | 0.03 | 0.03 | 1.08 | 0.0281 | 0.0683 |
|  | Asian | 0.02 | 0.05 | 0.01 | 1.02 | 0.6516 | 0.7385 |
|  | Black | -0.07 | 0.07 | -0.02 | 0.94 | 0.3215 | 0.3416 |
|  | Latinx | 0.03 | 0.04 | 0.01 | 1.03 | 0.4587 | 0.8825 |
|  | LGBTQ+ | 0.01 | 0.03 | 0.01 | 1.02 | 0.6665 | 0.8716 |
|  | First-gen | 0.04 | 0.03 | 0.02 | 1.04 | 0.2303 | 0.6525 |
|  | Financially unstable | -0.02 | 0.03 | -0.01 | 0.98 | 0.6173 | 0.7496 |
|  | STEM major | 0.04 | 0.04 | 0.02 | 1.04 | 0.3072 | 0.5286 |
|  | Lower division | 0.03 | 0.03 | 0.01 | 1.03 | 0.3716 | 0.4462 |
|  | GPA | -0.07 | 0.03 | -0.04 | 0.93 | 0.0237 | 0.2014 |
| The potential for personal technology issues (e.g., unstable internet connection) | Intercept | 0.29 | 0.14 | 0.44 | 1.34 | 0.0422 | NA |
|  | Woman | 0.13 | 0.04 | 0.06 | 1.14 | 0.0007 | 0.0059 |
|  | Asian | 0.03 | 0.05 | 0.01 | 1.03 | 0.6021 | 0.7385 |
|  | Black | -0.17 | 0.08 | -0.04 | 0.84 | 0.0224 | 0.0457 |
|  | Latinx | 0.02 | 0.04 | 0.01 | 1.02 | 0.6154 | 0.8825 |
|  | LGBTQ+ | 0.02 | 0.04 | 0.01 | 1.02 | 0.6434 | 0.8716 |
|  | First-gen | -0.05 | 0.04 | -0.03 | 0.95 | 0.1500 | 0.6377 |
|  | Financially unstable | 0.06 | 0.03 | 0.03 | 1.06 | 0.0904 | 0.1537 |
|  | STEM major | 0.05 | 0.04 | 0.02 | 1.05 | 0.2172 | 0.4616 |
|  | Lower division | -0.04 | 0.03 | -0.02 | 0.96 | 0.2111 | 0.3588 |
|  | GPA | 0.01 | 0.04 | 0.00 | 1.01 | 0.8582 | 0.9118 |
| Online monitored proctored testing | Intercept | 0.36 | 0.14 | 0.58 | 1.44 | 0.0108 | NA |
|  | Woman | 0.13 | 0.04 | 0.06 | 1.14 | 0.0005 | 0.0059 |
|  | Asian | -0.01 | 0.05 | 0.00 | 0.99 | 0.8272 | 0.8342 |
|  | Black | -0.17 | 0.07 | -0.04 | 0.85 | 0.0242 | 0.0457 |
|  | Latinx | 0.01 | 0.04 | 0.01 | 1.01 | 0.7493 | 0.9025 |
|  | LGBTQ+ | 0.03 | 0.04 | 0.01 | 1.03 | 0.5016 | 0.8716 |
|  | First-gen | 0.00 | 0.04 | 0.00 | 1.00 | 0.9328 | 0.9328 |
|  | Financially unstable | 0.08 | 0.03 | 0.04 | 1.09 | 0.0129 | 0.0548 |
|  | STEM major | 0.11 | 0.04 | 0.04 | 1.12 | 0.0084 | 0.1051 |
|  | Lower division | 0.01 | 0.03 | 0.01 | 1.02 | 0.6573 | 0.6573 |
|  | GPA | -0.01 | 0.04 | 0.00 | 0.99 | 0.8533 | 0.9118 |
| Struggling to have questions answered | Intercept | 0.48 | 0.14 | 0.49 | 1.62 | 0.0009 | NA |
|  | Woman | 0.10 | 0.04 | 0.04 | 1.11 | 0.0090 | 0.0256 |
|  | Asian | -0.06 | 0.05 | -0.02 | 0.94 | 0.2062 | 0.7374 |
|  | Black | -0.29 | 0.08 | -0.06 | 0.75 | 0.0001 | 0.0011 |
|  | Latinx | -0.10 | 0.04 | -0.04 | 0.91 | 0.0221 | 0.1881 |
|  | LGBTQ+ | 0.01 | 0.04 | 0.00 | 1.01 | 0.8944 | 0.9999 |
|  | First-gen | 0.04 | 0.04 | 0.02 | 1.04 | 0.3283 | 0.7972 |
|  | Financially unstable | 0.11 | 0.03 | 0.05 | 1.11 | 0.0022 | 0.0186 |
|  | STEM major | 0.03 | 0.04 | 0.01 | 1.03 | 0.4594 | 0.6047 |
|  | Lower division | 0.07 | 0.03 | 0.03 | 1.07 | 0.0421 | 0.1057 |
|  | GPA | -0.04 | 0.04 | -0.02 | 0.96 | 0.2395 | 0.5588 |
| Needing to talk with students who I don’t know during online group work | Intercept | 0.22 | 0.14 | 0.43 | 1.25 | 0.1260 | NA |
|  | Woman | 0.07 | 0.04 | 0.03 | 1.07 | 0.0622 | 0.1175 |
|  | Asian | 0.05 | 0.05 | 0.02 | 1.05 | 0.3109 | 0.7385 |
|  | Black | -0.17 | 0.08 | -0.04 | 0.85 | 0.0270 | 0.0459 |
|  | Latinx | -0.02 | 0.04 | -0.01 | 0.98 | 0.6628 | 0.8825 |
|  | LGBTQ+ | 0.08 | 0.04 | 0.03 | 1.08 | 0.0404 | 0.1718 |
|  | First-gen | 0.03 | 0.04 | 0.02 | 1.03 | 0.3865 | 0.8213 |
|  | Financially unstable | 0.02 | 0.03 | 0.01 | 1.02 | 0.4894 | 0.6400 |
|  | STEM major | 0.03 | 0.04 | 0.01 | 1.03 | 0.4141 | 0.6047 |
|  | Lower division | 0.09 | 0.03 | 0.04 | 1.09 | 0.0091 | 0.0514 |
|  | GPA | 0.01 | 0.04 | 0.01 | 1.01 | 0.7110 | 0.8634 |
| Nothing related to online courses makes my feelings of depression worse | Intercept | 0.20 | 0.07 | 0.06 | 1.22 | 0.0050 | NA |
|  | Woman | -0.02 | 0.02 | -0.01 | 0.98 | 0.2024 | 0.2294 |
|  | Asian | -0.02 | 0.02 | -0.01 | 0.98 | 0.5327 | 0.7385 |
|  | Black | 0.24 | 0.04 | 0.05 | 1.27 | 0.0000 | 0.0000 |
|  | Latinx | 0.00 | 0.02 | 0.00 | 1.00 | 0.8250 | 0.9025 |
|  | LGBTQ+ | -0.02 | 0.02 | -0.01 | 0.98 | 0.2806 | 0.6814 |
|  | First-gen | 0.01 | 0.02 | 0.00 | 1.01 | 0.6913 | 0.9328 |
|  | Financially unstable | -0.03 | 0.02 | -0.02 | 0.97 | 0.0444 | 0.1257 |
|  | STEM major | -0.01 | 0.02 | 0.00 | 0.99 | 0.5855 | 0.6635 |
|  | Lower division | -0.02 | 0.02 | -0.01 | 0.98 | 0.2714 | 0.4194 |
|  | GPA | -0.03 | 0.02 | -0.01 | 0.97 | 0.1426 | 0.5588 |
| Other | Intercept | 0.03 | 0.04 | 0.02 | 1.03 | 0.4756 | NA |
|  | Woman | 0.00 | 0.01 | 0.00 | 1.00 | 0.7769 | 0.8203 |
|  | Asian | -0.01 | 0.01 | 0.00 | 0.99 | 0.5605 | 0.7385 |
|  | Black | -0.02 | 0.02 | -0.01 | 0.98 | 0.2805 | 0.3179 |
|  | Latinx | 0.01 | 0.01 | 0.00 | 1.01 | 0.3479 | 0.8825 |
|  | LGBTQ+ | -0.01 | 0.01 | 0.00 | 0.99 | 0.5774 | 0.8716 |
|  | First-gen | 0.00 | 0.01 | 0.00 | 1.00 | 0.8030 | 0.9328 |
|  | Financially unstable | 0.02 | 0.01 | 0.01 | 1.02 | 0.0657 | 0.1396 |
|  | STEM major | 0.01 | 0.01 | 0.00 | 1.01 | 0.4980 | 0.6047 |
|  | Lower division | -0.02 | 0.01 | -0.01 | 0.99 | 0.1284 | 0.2425 |
|  | GPA | 0.00 | 0.01 | 0.00 | 1.00 | 0.6536 | 0.8547 |

**Table S6.** Summary of full result of the logistic regressions to identify demographic differences in the aspects of online college science courses that alleviate student depression. B represents unstandardized coefficients, whereas β represents standardized coefficients. OR represents the odds ratio (calculated as e^B^). Adjusted p-value is based on B-H corrections by demographic group across all aspects in the table. Focus categories are provided in the second column and reference groups are men, white, non-LGBTQ+, continuing generation, financially stable, non-STEM major, and upper division.

| **Aspect** | **Variable** | **B** | **SE B** | **β** | **OR** | ***p*** | **Adjusted *p*** |
| --- | --- | --- | --- | --- | --- | --- | --- |
| Being anonymous or being able to share my opinion without it being associated with my face | Intercept | 0.23 | 0.14 | 0.44 | 1.26 | 0.1016 | NA |
|  | Woman | 0.26 | 0.04 | 0.11 | 1.30 | 0.0000 | 0.0000 |
|  | Asian | 0.01 | 0.05 | 0.00 | 1.01 | 0.8347 | 0.8898 |
|  | Black | -0.10 | 0.07 | -0.02 | 0.90 | 0.1566 | 0.3147 |
|  | Latinx | 0.07 | 0.04 | 0.03 | 1.07 | 0.0994 | 0.2584 |
|  | LGBTQ+ | 0.05 | 0.04 | 0.02 | 1.06 | 0.1460 | 0.3796 |
|  | First-gen | 0.05 | 0.04 | 0.03 | 1.05 | 0.1497 | 0.5762 |
|  | Financially unstable | 0.03 | 0.03 | 0.02 | 1.03 | 0.3253 | 0.3957 |
|  | STEM major | 0.07 | 0.04 | 0.03 | 1.08 | 0.0668 | 0.8578 |
|  | Lower division | -0.05 | 0.03 | -0.02 | 0.95 | 0.1445 | 0.4697 |
|  | GPA | -0.02 | 0.03 | -0.01 | 0.98 | 0.5090 | 0.8272 |
| Getting questions answered | Intercept | 0.00 | 0.14 | 0.33 | 1.00 | 0.9762 | NA |
|  | Woman | 0.12 | 0.04 | 0.05 | 1.13 | 0.0006 | 0.0016 |
|  | Asian | 0.09 | 0.05 | 0.03 | 1.09 | 0.0699 | 0.3029 |
|  | Black | -0.03 | 0.07 | -0.01 | 0.97 | 0.6496 | 0.6496 |
|  | Latinx | 0.01 | 0.04 | 0.00 | 1.01 | 0.8672 | 0.9395 |
|  | LGBTQ+ | -0.04 | 0.04 | -0.02 | 0.96 | 0.2396 | 0.5122 |
|  | First-gen | 0.06 | 0.03 | 0.03 | 1.06 | 0.1098 | 0.5762 |
|  | Financially unstable | 0.09 | 0.03 | 0.04 | 1.09 | 0.0074 | 0.0968 |
|  | STEM major | 0.06 | 0.04 | 0.02 | 1.06 | 0.1421 | 0.8578 |
|  | Lower division | 0.03 | 0.03 | 0.01 | 1.03 | 0.3761 | 0.7795 |
|  | GPA | 0.03 | 0.03 | 0.01 | 1.03 | 0.3788 | 0.8207 |
| Clear communication with instructors | Intercept | -0.07 | 0.14 | 0.37 | 0.93 | 0.6106 | NA |
|  | Woman | 0.11 | 0.04 | 0.05 | 1.12 | 0.0025 | 0.0047 |
|  | Asian | 0.10 | 0.05 | 0.03 | 1.10 | 0.0461 | 0.3029 |
|  | Black | 0.05 | 0.07 | 0.01 | 1.05 | 0.4806 | 0.5206 |
|  | Latinx | 0.03 | 0.04 | 0.01 | 1.03 | 0.4142 | 0.6999 |
|  | LGBTQ+ | -0.03 | 0.04 | -0.01 | 0.97 | 0.3590 | 0.5185 |
|  | First-gen | 0.05 | 0.04 | 0.02 | 1.05 | 0.1773 | 0.5762 |
|  | Financially unstable | 0.07 | 0.03 | 0.04 | 1.07 | 0.0328 | 0.1193 |
|  | STEM major | 0.05 | 0.04 | 0.02 | 1.05 | 0.2109 | 0.8578 |
|  | Lower division | 0.00 | 0.03 | 0.00 | 1.00 | 0.9947 | 0.9947 |
|  | GPA | 0.07 | 0.04 | 0.03 | 1.07 | 0.0447 | 0.5816 |
| Easily getting help from instructors | Intercept | 0.08 | 0.13 | 0.31 | 1.09 | 0.5272 | NA |
|  | Woman | 0.14 | 0.04 | 0.06 | 1.15 | 0.0001 | 0.0004 |
|  | Asian | 0.06 | 0.05 | 0.02 | 1.06 | 0.1867 | 0.4044 |
|  | Black | -0.08 | 0.07 | -0.02 | 0.92 | 0.2256 | 0.3554 |
|  | Latinx | 0.00 | 0.04 | 0.00 | 1.00 | 0.9882 | 0.9882 |
|  | LGBTQ+ | -0.04 | 0.04 | -0.02 | 0.96 | 0.2758 | 0.5122 |
|  | First-gen | 0.02 | 0.03 | 0.01 | 1.02 | 0.5153 | 0.6797 |
|  | Financially unstable | 0.06 | 0.03 | 0.03 | 1.06 | 0.0595 | 0.1289 |
|  | STEM major | -0.02 | 0.04 | -0.01 | 0.98 | 0.6206 | 0.8611 |
|  | Lower division | -0.01 | 0.03 | -0.01 | 0.99 | 0.6655 | 0.9769 |
|  | GPA | 0.03 | 0.03 | 0.02 | 1.03 | 0.3492 | 0.8207 |
| Easily getting help from other students in class | Intercept | 0.21 | 0.12 | 0.24 | 1.23 | 0.0910 | NA |
|  | Woman | 0.09 | 0.03 | 0.04 | 1.09 | 0.0075 | 0.0109 |
|  | Asian | 0.08 | 0.04 | 0.03 | 1.09 | 0.0574 | 0.3029 |
|  | Black | -0.06 | 0.06 | -0.01 | 0.94 | 0.3458 | 0.4496 |
|  | Latinx | -0.03 | 0.04 | -0.01 | 0.97 | 0.4846 | 0.6999 |
|  | LGBTQ+ | -0.07 | 0.03 | -0.03 | 0.93 | 0.0377 | 0.2453 |
|  | First-gen | 0.06 | 0.03 | 0.03 | 1.06 | 0.0590 | 0.5762 |
|  | Financially unstable | 0.02 | 0.03 | 0.01 | 1.02 | 0.4448 | 0.4448 |
|  | STEM major | 0.01 | 0.04 | 0.00 | 1.01 | 0.7583 | 0.8611 |
|  | Lower division | 0.01 | 0.03 | 0.01 | 1.01 | 0.6775 | 0.9769 |
|  | GPA | -0.02 | 0.03 | -0.01 | 0.98 | 0.5087 | 0.8272 |
| Easily getting to know instructors | Intercept | 0.20 | 0.12 | 0.24 | 1.22 | 0.1153 | NA |
|  | Woman | 0.08 | 0.03 | 0.04 | 1.09 | 0.0124 | 0.0161 |
|  | Asian | 0.01 | 0.04 | 0.00 | 1.01 | 0.7711 | 0.8898 |
|  | Black | -0.14 | 0.06 | -0.03 | 0.87 | 0.0300 | 0.1301 |
|  | Latinx | -0.04 | 0.04 | -0.02 | 0.96 | 0.2794 | 0.6053 |
|  | LGBTQ+ | -0.07 | 0.03 | -0.03 | 0.93 | 0.0328 | 0.2453 |
|  | First-gen | 0.03 | 0.03 | 0.02 | 1.03 | 0.2889 | 0.6797 |
|  | Financially unstable | 0.03 | 0.03 | 0.01 | 1.03 | 0.3653 | 0.3957 |
|  | STEM major | -0.01 | 0.04 | -0.01 | 0.99 | 0.7243 | 0.8611 |
|  | Lower division | -0.03 | 0.03 | -0.01 | 0.97 | 0.3621 | 0.7795 |
|  | GPA | 0.00 | 0.03 | 0.00 | 1.00 | 0.8953 | 0.9407 |
| Easily getting to know other students in class | Intercept | 0.13 | 0.13 | 0.25 | 1.13 | 0.3161 | NA |
|  | Woman | 0.06 | 0.03 | 0.03 | 1.07 | 0.0586 | 0.0692 |
|  | Asian | 0.06 | 0.04 | 0.02 | 1.06 | 0.1784 | 0.4044 |
|  | Black | -0.08 | 0.07 | -0.02 | 0.93 | 0.2460 | 0.3554 |
|  | Latinx | -0.03 | 0.04 | -0.01 | 0.97 | 0.4470 | 0.6999 |
|  | LGBTQ+ | -0.03 | 0.03 | -0.01 | 0.97 | 0.3275 | 0.5185 |
|  | First-gen | 0.03 | 0.03 | 0.01 | 1.03 | 0.3906 | 0.6797 |
|  | Financially unstable | 0.06 | 0.03 | 0.03 | 1.06 | 0.0367 | 0.1193 |
|  | STEM major | -0.03 | 0.04 | -0.01 | 0.97 | 0.3959 | 0.8578 |
|  | Lower division | 0.02 | 0.03 | 0.01 | 1.02 | 0.4197 | 0.7795 |
|  | GPA | 0.02 | 0.03 | 0.01 | 1.02 | 0.6212 | 0.8966 |
| The flexibility of doing coursework when I want | Intercept | 0.73 | 0.14 | 0.65 | 2.09 | 0.0000 | NA |
|  | Woman | 0.06 | 0.04 | 0.03 | 1.07 | 0.0809 | 0.0876 |
|  | Asian | 0.07 | 0.05 | 0.02 | 1.07 | 0.1661 | 0.4044 |
|  | Black | -0.13 | 0.07 | -0.03 | 0.87 | 0.0644 | 0.2094 |
|  | Latinx | 0.01 | 0.04 | 0.01 | 1.01 | 0.7741 | 0.9148 |
|  | LGBTQ+ | -0.03 | 0.04 | -0.01 | 0.97 | 0.4213 | 0.5477 |
|  | First-gen | -0.02 | 0.04 | -0.01 | 0.98 | 0.5751 | 0.6797 |
|  | Financially unstable | 0.06 | 0.03 | 0.03 | 1.07 | 0.0532 | 0.1289 |
|  | STEM major | -0.01 | 0.04 | 0.00 | 0.99 | 0.8611 | 0.8611 |
|  | Lower division | -0.01 | 0.03 | -0.01 | 0.99 | 0.7515 | 0.9769 |
|  | GPA | -0.04 | 0.03 | -0.02 | 0.96 | 0.2434 | 0.7909 |
| The flexibility of doing coursework where I want | Intercept | 0.62 | 0.14 | 0.65 | 1.86 | 0.0000 | NA |
|  | Woman | 0.03 | 0.04 | 0.01 | 1.03 | 0.4322 | 0.4322 |
|  | Asian | 0.04 | 0.05 | 0.01 | 1.04 | 0.4310 | 0.8005 |
|  | Black | -0.18 | 0.07 | -0.04 | 0.84 | 0.0140 | 0.0912 |
|  | Latinx | 0.02 | 0.04 | 0.01 | 1.02 | 0.6590 | 0.8567 |
|  | LGBTQ+ | -0.02 | 0.04 | -0.01 | 0.98 | 0.5108 | 0.6037 |
|  | First-gen | -0.03 | 0.04 | -0.01 | 0.97 | 0.4074 | 0.6797 |
|  | Financially unstable | 0.03 | 0.03 | 0.02 | 1.03 | 0.3359 | 0.3957 |
|  | STEM major | 0.02 | 0.04 | 0.01 | 1.02 | 0.7008 | 0.8611 |
|  | Lower division | 0.00 | 0.03 | 0.00 | 1.00 | 0.9679 | 0.9947 |
|  | GPA | 0.00 | 0.04 | 0.00 | 1.00 | 0.9407 | 0.9407 |
| Having an instructor who appears to care about mental health | Intercept | 0.27 | 0.14 | 0.57 | 1.31 | 0.0600 | NA |
|  | Woman | 0.13 | 0.04 | 0.06 | 1.14 | 0.0005 | 0.0016 |
|  | Asian | -0.03 | 0.05 | -0.01 | 0.97 | 0.5905 | 0.8898 |
|  | Black | -0.11 | 0.07 | -0.02 | 0.90 | 0.1483 | 0.3147 |
|  | Latinx | 0.10 | 0.04 | 0.04 | 1.10 | 0.0213 | 0.2371 |
|  | LGBTQ+ | 0.07 | 0.04 | 0.03 | 1.07 | 0.0739 | 0.3202 |
|  | First-gen | -0.02 | 0.04 | -0.01 | 0.98 | 0.5655 | 0.6797 |
|  | Financially unstable | 0.06 | 0.03 | 0.03 | 1.06 | 0.0746 | 0.1386 |
|  | STEM major | 0.04 | 0.04 | 0.02 | 1.04 | 0.3500 | 0.8578 |
|  | Lower division | -0.06 | 0.03 | -0.03 | 0.94 | 0.0870 | 0.3771 |
|  | GPA | 0.05 | 0.04 | 0.02 | 1.05 | 0.2063 | 0.7909 |
| Being able to engage in an online science course without having to be seen | Intercept | 0.30 | 0.14 | 0.47 | 1.35 | 0.0367 | NA |
|  | Woman | 0.19 | 0.04 | 0.08 | 1.21 | 0.0000 | 0.0000 |
|  | Asian | -0.01 | 0.05 | 0.00 | 0.99 | 0.8898 | 0.8898 |
|  | Black | -0.10 | 0.07 | -0.02 | 0.90 | 0.1694 | 0.3147 |
|  | Latinx | 0.08 | 0.04 | 0.03 | 1.08 | 0.0729 | 0.2371 |
|  | LGBTQ+ | 0.06 | 0.04 | 0.03 | 1.06 | 0.1254 | 0.3796 |
|  | First-gen | 0.02 | 0.04 | 0.01 | 1.02 | 0.5274 | 0.6797 |
|  | Financially unstable | 0.05 | 0.03 | 0.02 | 1.05 | 0.1593 | 0.2588 |
|  | STEM major | 0.01 | 0.04 | 0.00 | 1.01 | 0.8378 | 0.8611 |
|  | Lower division | 0.01 | 0.03 | 0.00 | 1.01 | 0.8633 | 0.9947 |
|  | GPA | -0.01 | 0.04 | -0.01 | 0.99 | 0.7587 | 0.8966 |
| Nothing related to online courses helps me manage my depression | Intercept | 0.24 | 0.09 | 0.10 | 1.27 | 0.0049 | NA |
|  | Woman | -0.06 | 0.02 | -0.03 | 0.94 | 0.0048 | 0.0078 |
|  | Asian | -0.01 | 0.03 | 0.00 | 0.99 | 0.7423 | 0.8898 |
|  | Black | 0.13 | 0.04 | 0.03 | 1.13 | 0.0045 | 0.0586 |
|  | Latinx | -0.05 | 0.03 | -0.02 | 0.95 | 0.0583 | 0.2371 |
|  | LGBTQ+ | 0.00 | 0.02 | 0.00 | 1.00 | 0.9296 | 0.9296 |
|  | First-gen | 0.00 | 0.02 | 0.00 | 1.00 | 0.9910 | 0.9910 |
|  | Financially unstable | -0.04 | 0.02 | -0.02 | 0.96 | 0.0357 | 0.1193 |
|  | STEM major | 0.02 | 0.02 | 0.01 | 1.02 | 0.3896 | 0.8578 |
|  | Lower division | 0.04 | 0.02 | 0.02 | 1.04 | 0.0660 | 0.3771 |
|  | GPA | -0.03 | 0.02 | -0.01 | 0.97 | 0.1572 | 0.7909 |
| Other | Intercept | 0.02 | 0.03 | 0.01 | 1.02 | 0.5014 | NA |
|  | Woman | -0.03 | 0.01 | -0.01 | 0.97 | 0.0023 | 0.0047 |
|  | Asian | 0.00 | 0.01 | 0.00 | 1.00 | 0.8483 | 0.8898 |
|  | Black | 0.01 | 0.02 | 0.00 | 1.01 | 0.4628 | 0.5206 |
|  | Latinx | 0.02 | 0.01 | 0.01 | 1.02 | 0.0589 | 0.2371 |
|  | LGBTQ+ | 0.00 | 0.01 | 0.00 | 1.00 | 0.7109 | 0.7702 |
|  | First-gen | 0.00 | 0.01 | 0.00 | 1.00 | 0.6671 | 0.7227 |
|  | Financially unstable | 0.01 | 0.01 | 0.00 | 1.01 | 0.1919 | 0.2772 |
|  | STEM major | 0.00 | 0.01 | 0.00 | 1.00 | 0.6027 | 0.8611 |
|  | Lower division | -0.02 | 0.01 | -0.01 | 0.98 | 0.0040 | 0.0517 |
|  | GPA | 0.00 | 0.01 | 0.00 | 1.00 | 0.7113 | 0.8966 |
